# Investigating the effects of external fields polarization on the coupling of pure magnetic waves in the human body in very low frequencies

**DOI:** 10.1186/1477-044X-5-3

**Published:** 2007-05-15

**Authors:** Laleh Golestani-Rad, Behzad Elahi, Jalil Rashed-Mohassel

**Affiliations:** 1 Laboratory of Electromagnetics and Acoustics (LEMA), Ecole Polytechnique Fédérate de Lausanne (EPFL), Switzerland; 2 School of Medicine, Tehran University of Medical Sciences, Tehran, Iran; 3 Center of Excellence on Applied Electromagnetic Systems, Department of Electrical and Computer Engineering, Faculty of Engineering, University of Tehran, Iran

## Abstract

In this paper we studied the effects of external fields' polarization on the coupling of pure magnetic fields into human body. Finite Difference Time Domain (FDTD) method is used to calculate the current densities induced in a 1 cm resolution anatomically based model with proper tissue conductivities. Twenty different tissues have been considered in this investigation and scaled FDTD technique is used to convert the results of computer code run in 15 MHz to low frequencies which are encountered in the vicinity of industrial induction heating and melting devices. It has been found that external magnetic field's orientation due to human body has a pronounced impact on the level of induced currents in different body tissues. This may potentially help developing protecting strategies to mitigate the situations in which workers are exposed to high levels of external magnetic radiation.

## Background

Coupling of external electromagnetic fields into the human body has been subject of many investigations in recent years especially with several epidemiological studies linking higher rates of incidence of certain cancers with electromagnetic radiation [1,2]. Many investigations have been made to evaluate EM waves coupling into different organs of body and many international guidelines and standards have been set up for exposure limits in order to protect workers against nonionizing radiation in their workplaces.

Regarding the fact that many cancer associations have referred to magnetic radiation and considering the fact that magnetic fields are not shielded by conventional shielding structures, has led to a dominant interest in exploring potential hazards of magnetic induction.

There are many studies in recent years evaluating levels of current densities induced in different body tissues when exposed to low frequency EM waves. The well known scaled FDTD technique has been used widely since proposed by Gandhi [3-5] to convert the results of a simulation performed in a higher frequency (in the range of mega hertz) to the results of very low frequency coupling caused by power line radiation in 50 and 60 Hz. Other studies considering the effects of uniform and nonuniform magnetic fields with various polarizations are also performed at 60 Hz using quasi-static impedance method [6].

Different combinations of incident waves, including pure magnetic fields with different polarizations and both electric and magnetic fields in the form of a uniform plane wave have been studied in Gandhi's works at power line frequencies. Other studies have explored effects of very low frequency pure electric fields using high resolution models (with cubic voxels of 3.6 mm edges) [6]. Industrial frequencies in the range of kilo hertz have been also studied due to potential risks imposed on workers near induction heating and melting devices and it has also been shown that for low frequency dosimetric applications, using 1 cm resolution model with realistic shape but relatively lower resolution in discrimination of internal tissues may give good results with acceptable accuracy [7].

In this paper a 1 cm resolution anatomically based model with 20 different organs/tissues has been used to evaluate current densities induced in different parts of body when exposed to pure magnetic fields in frequency of 1 KHz. This particular frequency is of interest for two major reasons: first, this is the frequency mostly radiated by industrial heating and melting devices in work places for which many basic restrictions indicating the maximum permissible values of electric and magnetic field inside the human body have already been established. The second, this is the frequency proven to have a remarkable impact on the phenomenon of cell electroporation [8,9]. In this phenomenon, tiny pores will be formed on the membrane of cells exposed to electric fields with a particular frequency and magnitude. These pores are formed in several microseconds and may last up to some seconds and the process is irreversible if the voltage induced on the cell membrane exceeds a critical value and cell death will occur. Therefore, studying the magnitude of induced electric fields in different body tissues may potentially help obtaining a better control and insight into potential occurrence of electroporation phenomenon.

The model is manually prepared by converting MRI images of a 46-year old man with the height of 178 cm into suitable matrixes of electrical properties of computational space to be used in FDTD algorithm. MRI images were T1-weighted and obtained with a 1.5T unit and the following parameters: TR = 300–450 ms, TE = 12–15 ms, matrix size = 256 × 256.

20 different tissues and related organs have been identified by an expert physician and a consulting radiologist and have been discriminated due to variations in their conductivities. Pure magnetic radiation is simulated using two plane waves traveling in opposite directions with electric components canceling each other.

It is shown that the orientation of magnetic vector in respect with the body may have a considerable impact on the coupling of external fields into different organs. This may be useful to be considered when seeking for appropriate protective strategies against unwanted effects of external radiation.

## FDTD algorithm and human body modeling

**Figure 1:**
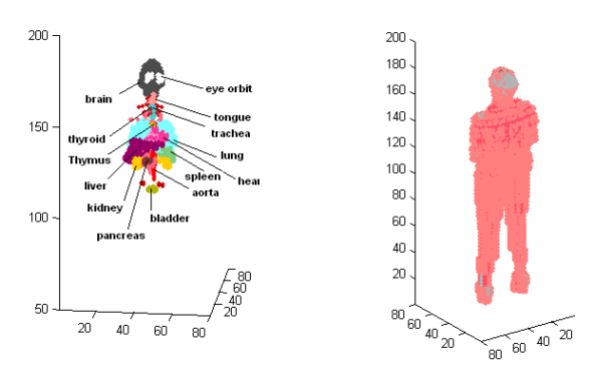
Views of anatomic model of human body used in the simulation.

The anatomic based human body model used in our work has been prepared manually using data from MRI images of a 46-year old man with the height of 178 cm. These images have been then converted to thee dimensional matrixes introducing electrical properties of human body to the computational environment in FDTD algorithm. Fig. 1 shows some sensitive organs included in the model. Related conductivities for different parts of body are obtained from [10] for the frequency of 1 KHz.

The whole FDTD computational space has been divided into 80 × 80 × 200 = 1280000 cubic cells with the cell size of 1 cm. The human body is suspended in the air and the computational space is terminated to a 10-layer PML (Perfectly Matched Layer) with a grading profile and the optimum conductivity as described in [11].

To simulate the interaction of low frequency incident waves with human body, scaled FDTD method is used as described in [4]. According to Gandhi, in this case the electric fields outside the body depend not on the internal tissue properties, but only on the shape of the body as long as the quasi-static approximation is valid, i.e., the size of the body is a factor of 10 or more smaller the wavelength, and |*σ *+ *jωε*| >> *ωε*_0 _where *σ *and *ε *are the conductivity and permittivity of the tissues, respectively. Under these conditions electric fields in air are normal to the body surface and the internal electric fields are given from the boundary conditions in terms of the fields outside:

(1) $$j\omega \varepsilon_{0}\overset{⌢}{n}.{\vec{E}}_{air}=(\sigma +j\omega \varepsilon )\overset{⌢}{n}.{\vec{E}}_{tissue}$$

A higher quasi-static frequency of *f' *maybe therefore used for irradiation of the model and the induced electric fields *E' *thus calculated may be scaled back to the frequency of interest *f*.

From the equation (1) we can write:

(2) $${\vec{E}}_{Tissue}(f)=\frac{\omega }{\omega \prime }\frac{\left(\sigma \prime +j\omega \varepsilon \prime \right)}{\left(\sigma +j\omega \varepsilon \right)}{\vec{E}}_{Tissue}^{\prime }(f\prime )≅\frac{f\sigma \prime }{f\prime \sigma }{\vec{E}}_{Tissue}^{\prime }(f\prime )$$

Assuming that *σ *+ *jωε *≅ *σ *at both *f *and *f'*

In many cases if *σ' *and *σ *are close enough we can simplify the equation (2) to

(3) $$E(r)=\frac{f}{f^{\prime }}E^{\prime }(r)$$

In our program the actual FDTD program is performed at a frequency of *f' *= 15 *MHz *and afterwards the induced electric field *E' *in the frequency of *f' *is scaled to the frequency of interest *f *using equation (3). As it is obvious from (2) the permittivity of tissues doesn't affect the results significantly, therefore the value of *ε*_*r *_= 1 has been set for the whole environment to hasten the speed of wave propagation.

In order to generate a homogeneous magnetic field the model is exited simultaneously by two plane waves traveling in opposite directions. With the appropriate orientation of plane waves electric field components cancel out and the magnetic components superpose constructively. Two different orientations for magnetic field vector are studied: front to back and foot to head and the magnitude of electric field of each plane wave is 100 V/m.

The total average electric current for different layers of body height is obtained from the equation (4). When FDTD runs, the magnitude of electric fields inside the body reaches an overshoot during the beginning time steps and then follows a sinusoidal pattern with lower amplitude. This overshoot has not been considered when calculating the temporal maximum of currents. To calculate the average of total electric current for each layer we have simply added elemental currents of each cell in the layer and divided the result by the number of contributing cells.

(4) $${\left|J_{total}\right|}_{k}=\sum_{i,j}\sigma_{i,j,k}{\left[\sum_{m=x,y,z}{\left|E_{m}^{i,j,k}\right|}^{2}\right]}^{\frac{1}{2}}$$

## Numerical results

**Figure 2:**
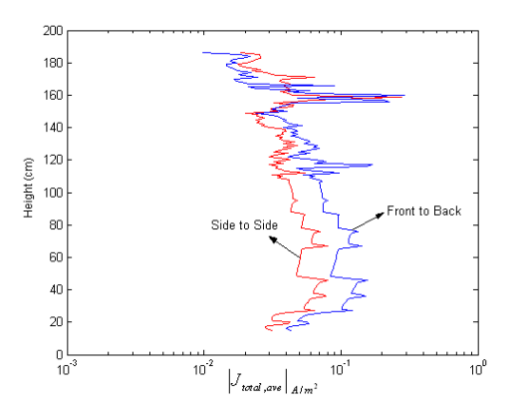
Layer-averaged electric current for two different polarizations of magnetic field.

Fig. 2 shows the layer-averaged induced currents computed for two different polarizations of magnetic field. It is obvious that orientation of the body with respect to external fields has a pronounced impact on the coupling of fields into body.

**Figure 3:**
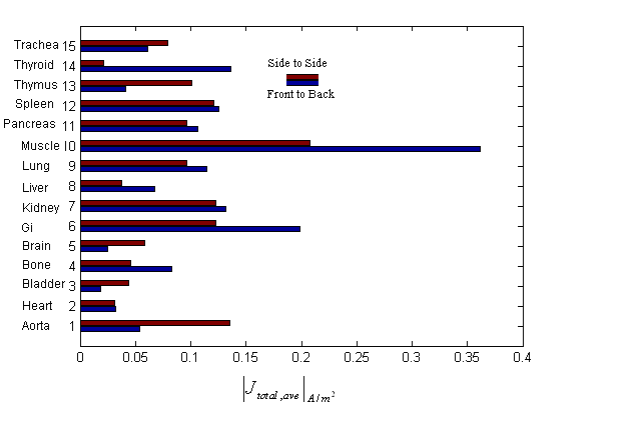
Organ-averaged electric current for different parts of body.

For different organs, the organ-averaged electric current has been also calculated for two magnetic field's polarizations. Fig. 3 shows the results of this calculation. It is observed that for most of organs the front to back magnetic vector orientation has more powerful coupling effects than side to side magnetic vector, though this trend is reversed for some specific parts of body. In [4] also, different orientations of fields due to human body are studied for 60 Hz pure magnetic fields, but the results do not show such pronounced differences between curves of layer averaged electric currents. This may be because of the fact that in [4] a more homogeneous human model is used and less discrete tissue/organs are considered.

The differences are more pronounced for some specific organs like thymus and thyroid glands. If it is clinically proven that electric currents may some how affect the function of these organs, then the results obtained from this study may help suggesting some mitigating techniques to reduce the potential hazardous effects of unwanted external radiation, i.e. by setting some restrictions on workers orientations due to industrial devices.

**Figure 4:**
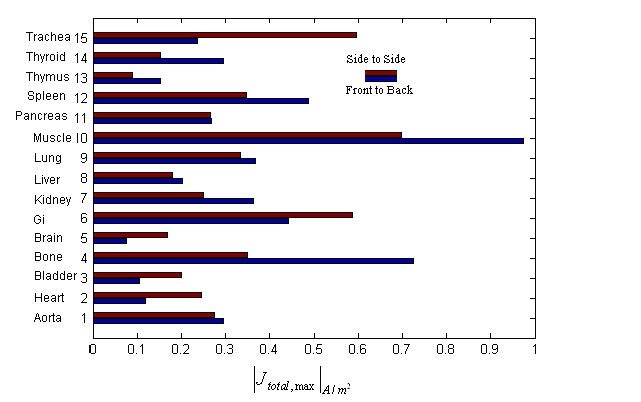
Maximum of total electric current for different parts of body.

To verify the model, the results are compared with the results presented in [7]. In this work, a pure magnetic field with the vector oriented from front to back of the body is simulated to produce *B *= 30.7 *μT*. The electric field of each of the two plane waves must therefore be 0.92 V/m. Fig. 4 shows the maximum of electric current induced in different body parts. If we scale the results presented in Fig. 4 by dividing them to the scale factor 1087, they will show a good agreement with results presented in [7]. Slight differences between results may be due to differences in weight and height of actual models.

## Conclusion

In this work the effects of external magnetic fields' orientation on the coupling of fields into human body in very low frequencies have been studied using an inhomogeneous anatomic human model. It has been shown that the coupling of external fields into different organs and tissues may be affected considerably by changing the orientation of external filed vectors. This effect is more pronounced for some specific organs like thyroid and thymus gland and calls for further studies on potential effects of electric currents on function of these organs.

**Table 1:** Electrical properties of different body organs/tissues in

Organ/Tissue	Conductivity in 1000 Hz
Aorta	0.26
Lung	0.15
Bone	0.05
Kidney	0.11
Liver	0.04
Heart	0.11
Atrium	0.11
Artery	1.5
Skin	0.02
Bladder	0.2
Brain	0.075
Trachea	0.3
Tongue	0.27
Thyroid	0.52
Spleen	0.1
Gi	0.52
Vein	1
Pancreas	0.52
Muscle	0.32
Eye orbit	0.5

## References

[B1] Sahl JD Kelsh MA Greenland S Cohort and nested case-control studies of hematopoietic cancers and brain cancer among electric utility workers Epidemiology 1993 5 104 114 10.1097/00001648-199303000-00005 8452898

[B2] Savitz DA Loomis DP Magnetic field exposure in relation to leukemia and brain cancer mortality among electric utility workers American Journal of Epidemiology 1995 5 123 134 7817968 10.1093/oxfordjournals.aje.a117400

[B3] Furse CM Gandhi OP Calculation of electric fields and currents induced in a millimeter-resolution human model at 60 Hz using the FDTD method Journal of Bioelectromagnetics 1998 5 293 299 10.1002/(SICI)1521-186X(1998)19:5<293::AID-BEM3>3.0.CO;2-X 9669543

[B4] Gandhi OP Chen JY Numerical dosimetry at power-line frequencies using anatomically based models Bioelectromagnetics 1992 Suppl 1 43 60 10.1002/bem.2250130706 1285721

[B5] Gandhi OP Kang G Wu D Lazzi G Currents Inducedin Anatomic Models of the Human for Uniformand Nonuniform Power Frequency Magnetic Fields Bioelectromagnetics 2001 5 112 121 10.1002/1521-186X(200102)22:2<112::AID-BEM1014>3.0.CO;2-0 11180257

[B6] Dawson TW Stuchly MA High-resolution organ dosimetry for human exposure to low-frequencymagnetic fields IEEE transactions on magnetics 1998 5 708 718 10.1109/20.668071

[B7] Gustrau F Bahr A Rittweger M Goltz S Eggert S Simulation of induced current densities in the human body at industrial induction heating frequencies IEEE Transactions on Electromagnetic Compatibility 1999 5 480 486 10.1109/15.809851

[B8] DeBruin KA Krassowska W Modeling Electroporation in a Single Cell. I. Effects of Field Strength and Rest Potential Biophysical Journal 1999 5 1213 1224 10465736 10.1016/S0006-3495(99)76973-0 PMC1300413

[B9] Weaver JC Chizmadzhev YA Theory of electroperation: A review Bioelectrochemistry and Bioenergetics 1996 5 135 160 10.1016/S0302-4598(96)05062-3

[B10] Taflove A Hagness SC Computational Electrodynamics: The Finite-difference Time-domain Method 2000 Artech House Press

[B11] Berenger JP A perfectly matched layer for the absorption of electromagnetic waves Journal of computational physics 1994 5 185 200 10.1006/jcph.1994.1159

